# First Detection of the SARS-CoV-2 Omicron BA.5/22B in Monaco

**DOI:** 10.3390/microorganisms10101952

**Published:** 2022-09-30

**Authors:** Philippe Colson, Christian Lavagna, Jérémy Delerce, Guillaume Groshenry, Nouara Yahi, Jacques Fantini, Bernard La Scola, Thomas Althaus

**Affiliations:** 1 IHU Méditerranée Infection, 19-21 Boulevard Jean Moulin, 13005 Marseille, France; 2 Institut de Recherche pour le Développement (IRD), Aix-Marseille University, Microbes Evolution Phylogeny and Infections (MEPHI), 27 boulevard Jean Moulin, 13005 Marseille, France; 3 Assistance Publique-Hôpitaux de Marseille (AP-HM), 264 rue Saint-Pierre, 13005 Marseille, France; 4 Centre Scientifique de Monaco, 8 Quai Antoine 1er, 98000 Monaco, Monaco; 5 INSERM UMR S 1072, Aix-Marseille Université, 13005 Marseille, France; 6 Direction de l’Action Sanitaire, 48 Boulevard d’Italie, 98000 Monaco, Monaco

**Keywords:** SARS-CoV-2, variant, lineage, Omicron, BA.5, 22B, Monaco

## Abstract

The Omicron BA.5/22B variant has been designated as a “variant of concern” by the World Health Organization. We describe, here, the first evidence in Monaco of infection with an Omicron BA.5/22B variant, probably imported from the Republic of Seychelles, harboring a rare combination of non-BA.5/22B signature amino acid changes. SARS-CoV-2 neutralizing antibodies were measured with a surrogate virus neutralization test. SARS-CoV-2 genotype screening was performed on nasopharyngeal samples with a multiplex qPCR assay. The SARS-CoV-2 genome was obtained by next-generation sequencing with the Illumina COVID-seq protocol, then assembly using bioinformatics pipelines and software was performed. The BA.5/22B spike protein structure was obtained by molecular modeling. Two spouses were SARS-CoV-2-diagnosed the day they returned from a one-week trip in the Republic of Seychelles. SARS-CoV-2 qPCR screening for variant-specific mutations identified an Omicron variant BA.1/21K, BA.4/22A, or BA.5/22B. A SARS-Co-2 BA.5/22B variant genome was recovered from one of the spouses. Aside from BA.5/22B-defining amino acid substitutions, four other amino acid changes were encoded including Q556K in ORF1a, K2557R in ORF1b, and A67V and A829T in spike; only 13 genomes in sequence databases harbored these four mutations concurrently. Structural analysis of this BA.5/22B variant predicted that A829T in spike may result in a compaction that may affect conformational plasticity. Overall, our findings warrant performing genome-based genotypic surveillance to survey accurately the emergence and circulation of SARS-CoV-2 variants worldwide and point out that their first occurrence in a country is often through international travel despite implemented countermeasures.

## 1. Introduction

The SARS-CoV-2 pandemic has been punctuated by distinct outbreaks linked to different variants since the summer of 2020 [1,2,3]. Some of these variants remained epidemic, restricted to a relatively limited geographical area, while others became pandemic, for reasons which are not precisely elucidated. The pandemic variants in 2022 have been the Omicron variants BA.1 (Pangolin classification (https://cov-lineages.org/pangolin.html; accessed on 12 July 2022) [3])/21K (Nextclade classification ((https://clades.nextstrain.org/; accessed on 11 July 2022) [4,5]) and BA.2/21L (https://covariants.org; accessed on 19 July 2022) [6,7]. They emerged in France at the end of November 2021 and December 2021, respectively, and replaced the Delta variant. Recently, a lull in the incidence of SARS-CoV-2 infections occurred in Western Europe (https://coronavirus.jhu.edu/map.html; accessed on 11 July 2022). The variants currently particularly watched are Omicron BA.4/22A and BA.5/22B, which are designated as “variants of concern” (VOC) by the World Health Organization (https://www.who.int/activities/tracking-SARS-CoV-2-variants; accessed on 11 July 2022). They have enlarged the set of VOCs previously comprised by the Alpha (B.1.1.7), Beta (B.1.351), Gamma (P.1), and Delta (B.1.617.2) variants.

The Omicron lineage encompasses several variants that successively or concomitantly circulated worldwide, including BA.1/21K, BA.2/21L, BA.3, BA.4/22A, BA.5/22B, BA.2.12.1/22C, and BA.2.75/22D (https://covariants.org; accessed on 19 July 2022) [6,8,9]. The Omicron BA.1/21K variant emerged in November 2021, with early sequences being predominantly from South Africa. It harbored 37 nucleotide and amino acid mutations in its spike protein, which was dramatically more than for previous variants, and 72 along the whole genome, compared to the Wuhan-Hu-1 isolate (genome GenBank accession no. NC_045512.2). The Omicron BA.2/21L variant arose in late 2021 and early 2022, and replaced the Omicron BA.1/21K variant worldwide during the first months of 2022. Its genome harbored 76 nucleotide and amino acid mutations, including 38 nucleotide and amino acid mutations (21 amino acid mutations in the spike) in common with the Omicron BA.1/21K variant. The Omicron BA.4/22A and BA.5/22B variants arose in late 2021 and in early 2022, respectively, possibly in South Africa where early sequences were predominantly detected. They replaced the Omicron BA.2/21L variant worldwide during summer 2022. Their genomes harbored 111 and 105 nucleotide and amino acid mutations, respectively, and their spike proteins were identical between each other and share many mutations with those of the Omicron BA.1/21K and BA.2/21L variants. Particularly, amino acid substitutions L452R and F486V and deletions H69- and V70- are present in the spike of the Omicron BA.4/22A and BA.5/22B variants while amino acid substitution Q493R is absent. The Omicron BA.3 variant has had a very limited spread with only a few hundred genomes available globally. The Omicron BA.2.12.1/22C variant (109 nucleotide and amino acid substitutions) appears to have arisen in late 2021 or early 2022 in North America, where it mostly circulated thereafter. The Omicron BA.2.75/22D variant (118 nucleotide and amino acid substitutions) appears to have arisen in late spring 2022 in India, where it mostly spread thereafter, albeit genomes have been obtained since then in many countries worldwide. These Omicron BA.2.12.1/22C and BA.2.75/22D variants share many spike mutations with the Omicron BA.1/21K and BA.2/21L variants. Regarding the Omicron BA.5/22B variant, it was reported to have a growth advantage over the Omicron BA.1 and BA.2 variants [10]. As for the case of the Omicron BA.4/22A variant, it was reported to exhibit considerable escape to neutralizing immunity elicited by BA.1 infection [11] and to be resistant to most broad neutralizing antibodies, although it could be neutralized by some therapeutic antibodies [12] This escape to neutralizing antibodies may be explained by the D405N and F486V mutations in the spike protein, while the S371F, D405N, and R408S mutations within spike may also play a role. The spike F486V mutation was reported to facilitate escape from neutralizing antibodies but also to jeopardize the affinity of the viral spike for the angiotensin-converting enzyme 2 (ACE2) viral receptor [10]. The spike R493Q reversion mutation was reported to restore this affinity for the receptor and consequently to enhance the viral fitness of the Omicron BA.5/22B variant [10]. Additionally, Omicron variants have been involved in the generation of various SARS-CoV-2 recombinants, with previous VOCs as for the case of hybrids of Delta and Omicron BA.1/21K genomes [13], but also between each other [14].

It has been possible to observe in the Marseille geographical area that in the vast majority of cases, the emergence of a new SARS-CoV-2 variant was due to its introduction from abroad in a context of travel, with transport identified by cars, boats, trains, or planes [1]. This points out the limits in the performance of the means implemented to hamper the importation of variants from one country to another through international travel [15]. We describe, here, the first evidence in Monaco of infection with an Omicron BA.5/22B variant, probably imported from the Republic of Seychelles, harboring a rare combination of non-signature amino acid changes.

## 2. Materials and Methods

### 2.1. Diagnosis of SARS-CoV-2 Infection by Real-Time Reverse Transcription-PCR

Nasopharyngeal samples were tested for the presence of SARS-CoV-2 RNA by real-time reverse transcription-PCR (qPCR) using the Cobas SARS-CoV-2 kit used on the Cobas 6800 system (Cobas 6800; Roche Diagnostics, Mannheim, Germany), according to the manufacturer’s instructions.

### 2.2. SARS-CoV-2 Serology and SARS-CoV-2 Seroneutralisation Assay

The Monaco government has implemented a national public health program including free SARS-CoV-2 RT-PCR testing, vaccination, and serological services in a community center. Regarding the serological service, a systematic individual surveillance has been offered to all residents every 6 months, which included serological assessment of anti-nucleocapsid (anti-N) antibodies, anti-spike receptor binding domain (RBD) antibodies, and neutralizing antibodies. The rationale for this program was to inform all residents about their past exposure to SARS-CoV-2 and their level of protection against severe forms of COVID-19.

Anti-RBD and anti-N total binding antibody titers were assessed by the Elecsys anti-SARS-CoV-2 S immunoassay on a Roche Cobas 6000 (Roche Diagnostics) according to the manufacturer’s instructions. The analyte concentration of each sample was expressed in U/mL and E/S for anti-RBD and anti-N total binding antibodies, respectively [16]. SARS-CoV-2 neutralizing antibodies (nAbs) against the Wuhan-Hu-1 strain and the B.A.1.1.529 (Omicron) variant were measured with the GenScript kit (cPass, Piscataway, NJ, USA), a surrogate virus neutralization test (sVNT) whose accuracy was reported elsewhere and results were expressed as inhibition rate (%) and in IU/mL based on WHO international standards [17,18].

### 2.3. Reverse Transcription PCR-Based SARS-CoV-2 Genotyping

SARS-CoV-2 genotype screening was performed on nasopharyngeal samples with a multiplex qPCR assay using the Cobas SARS-CoV-2 Variant Set 1 test (RUO) (Roche Diagnostics) on the automated Cobas 6800 system. It consisted in the detection of mutations E484K, N501Y, and deletion HV-69/70, according to the manufacturer’s instructions.

### 2.4. SARS-CoV-2 Genome Sequencing and Analysis

SARS-CoV-2 genomes were obtained and analyzed as described previously [1,19]. Briefly, next-generation sequencing was carried out with the Illumina COVID-seq protocol on the NovaSeq 6000 instrument (Illumina Inc., San Diego, CA, USA). Sequence read processing and genome analysis were performed as described previously [1,19]. Briefly, base calling was carried out with the Dragen Bcl Convert pipeline (v3.9.3; https://emea.support.illumina.com/sequencing/sequencing_software/bcl-convert.html; accessed on 11 July 2022; (Illumina Inc.)), mapping was carried out by the bwa-mem2 tool (v2.2.1; https://github.com/bwa-mem2/bwa-mem2; accessed on 11 July 2022) on the Wuhan-Hu-1 isolate genome (GenBank accession no. NC_045512.2) before cleaning using the SAMtools program (v. 1.13; https://www.htslib.org/; accessed on 11 July 2022) [20]. Variant calling was performed by FreeBayes (v1.3.5; https://github.com/freebayes/freebayes; accessed on 11 July 2022) [21], and consensus genomes were built using the Bcftools program (v1.13; https://samtools.github.io/bcftools/bcftools.html; accessed on 11 July 2022). Nucleotide and amino acid changes compared to the Wuhan-Hu-1 genome were identified with the Nextclade tool (https://clades.nextstrain.org/; accessed on 11 July 2022) [4,5]. Nextstrain clades and pangolin lineages were determined with the Nextclade web application (https://clades.nextstrain.org/; accessed on 11 July 2022) [4,5] and the pangolin tool (https://cov-lineages.org/pangolin.html; accessed on 11 July 2022) [22], respectively. Phylogeny was reconstructed by the IQTree (v2.1.3; http://www.iqtree.org/; accessed on 11 July 2022) [23] following sequence alignment with MAFFT (https://mafft.cbrc.jp/alignment/server/; accessed on 11 July 2022) [24], and trees were visualized with MEGA X [25]. The genomes most similar to the sequence obtained here, among those of the entire EpiCoV database (https://www.gisaid.org/; accessed on 11 July 2022) [26,27], were selected through a search with the AudacityInstant tool (https://www.epicov.org/epi3/frontend#4adee9; accessed on 11 July 2022) [26,27], then incorporated in the phylogenies together with the Wuhan-Hu-1 genome sequence.

### 2.5. SARS-CoV-2 Spike Structure Analyses

The structure of the BA.5/22B spike protein was obtained by molecular modeling as previously described [28]. Briefly, a complete structure of the spike protein was generated from the original 20B strain (Wuhan Hu-1 isolate with the D614G substitution in the spike protein, Protein Data Bank (pdb) number 7BNM (doi: 10.2210/pdb7BNM/pdb) [29]. All gaps in the PDB file were fixed by inserting the missing amino acids with Robetta (https://robetta.bakerlab.org; accessed on 1 July 2022) [30]. The structure was then submitted to several rounds of energy minimization with the Polak-Robière algorithm as described previously. This source file model was used to introduce the specific mutational profiles of BA.5/22B, which was then simulated with the same method. 

## 3. Results

### 3.1. Cases’ Reports

The index case was a 47-year-old man who was diagnosed with SARS-CoV-2 by qPCR (Cycle threshold value, 18) on 19/04/2022, and lives in the Principality of Monaco (Monaco), a 2.1 km^2^ city state with approximately 38,000 residents located on the Mediterranean Sea in the French territory close to the Italian border. This patient returned two days before being SARS-CoV-2-diagnosed from a one-week family trip in the Republic of Seychelles, an archipelagic island country in the Indian Ocean with approximatively 98,000 inhabitants. The first symptom was ageusia and it occurred the day of the return to Monaco. At the time of diagnosis, 2 days later, the patient was exhibiting fever at 38 °C, asthenia, and mild respiratory symptoms. Clinical symptoms vanished 2 days later. This patient was fully vaccinated (3 doses) against SARS-CoV-2 with the Pfizer-BioNTech COVID-19 mRNA vaccine. The index case’s spouse, also fully vaccinated, was similarly diagnosed with SARS-CoV-2 on a nasopharyngeal sample collected on 19 April 2022.

### 3.2. SARS-CoV-2 Serology and Seroneutralization Assay

Regarding the index case, an initial serology was carried out on a sample collected on 16 November 2021, with anti-N antibodies measured at 0.07 E/S and anti-RBD measured at 360 U/mL, while the anti-Wuhan Hu-1 nAb titer was 67 IU/mL and anti-Omicron nAbs were not detected. A second serology was carried out on 12/05/2022, three weeks post-SARS-CoV-2 diagnosis, with anti-N measured at 5.87 E/S, anti-RBD measured at 46,949 U/mL, while the anti-Wuhan Hu-1 nAb titer was 927 IU/mL and the anti-Omicron nAb titer was 950 IU/mL. Regarding the index case’s spouse, anti-N antibodies were measured at 0.06 E/S on 16/11/2021, with anti-RBD antibodies measured at 429 U/mL, while the Wuhan Hu-1 nAb titer was 97.1 IU/mL and anti-Omicron nAbs were not detected. As for the index case, a second serology was carried out on 12 May 2022, with anti-N measured at 7.91 E/S, anti-RBD at 19,481 U/mL, while the anti-Wuhan Hu-1 nAb titer was 530 IU/mL and the anti-Omicron nAb titer was 12 IU/mL.

### 3.3. SARS-CoV-2 Genotyping

**Figure 1:**
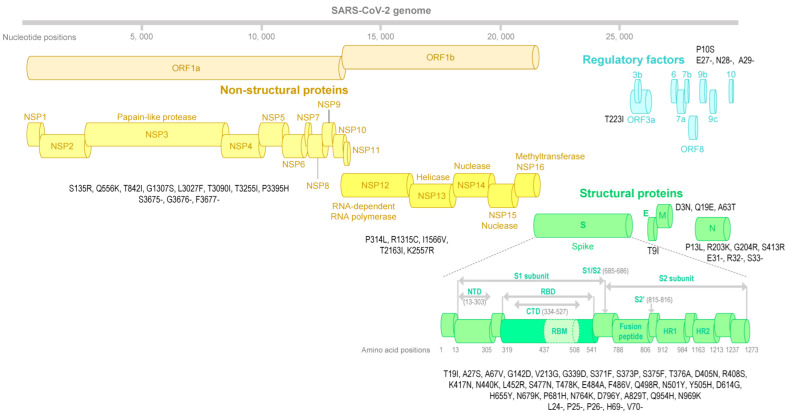
Map of the SARS-CoV-2 Omicron BA.5/22B genome obtained here and its amino acid mutations. NTD = N-terminal domain; RBD = receptor binding domain.

**Figure 2:**
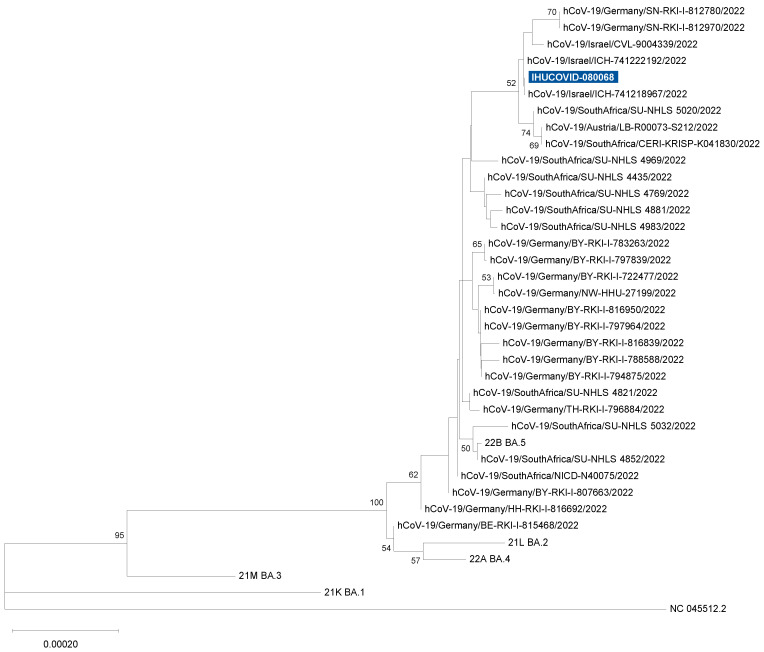
Phylogeny reconstruction based on SARS-CoV-2 Omicron 22B/BA.5 genomes. The SARS-CoV-2 genome obtained, here, is indicated by a white bold font and a blue background. The phylogenetic tree was built with IQTree (v2.1.3; http://www.iqtree.org/; accessed on 11 July 2022) [23] after sequence alignment with MAFFT (https://mafft.cbrc.jp/alignment/server/; accessed on 11 July 2022) [24], and trees were visualized with MEGA X [25].

SARS-CoV-2 qPCR screening for variant-specific mutations showed positivity for spike deletion 69–70 and spike mutation N501Y, and negativity for spike mutation E484K. This pattern was indicative of an Omicron variant BA.1/21K, BA.4/22A, or BA.5/22B. The sample from the index case’s spouse showed the same reactivities. Next-generation genome sequencing allowed obtaining a full-length genome that harbored all signature nucleotide substitutions and deletions of the Omicron BA.5/22B variant (Figure 1) and was identified as of this genotype by the Nextclade and Pangolin tools, and by the phylogenetic analysis (Figure 2).

The genome sequence obtained and analyzed, here, was deposited in the NCBI GenBank nucleotide sequence database (https://www.ncbi.nlm.nih.gov/genbank/; accessed on 11 August 2022) [31] (Accession no. ON989858), and on the IHU Méditerranée Infection website (https://www.mediterranee-infection.com/tout-sur-le-coronavirus/sequencage-genomique-sars-cov-2/; accessed on 11 July 2022; no. IHUCOVID-080068). Aside from BA.5/22B-defining amino acid substitutions, four other amino acid changes were encoded including Q556K in ORF1a gene product, K2557R in ORF1b gene product, and A67V and A829T in the spike protein. CovSPECTRUM (https://cov-spectrum.org/; accessed on 11 July 2022) detected (as of 13 June 2022) 1656, 16, 9, and 7 BA.5 genomes that harbored these mutations, respectively. The sequenced genomes that first harbored these mutations were obtained from patients sampled on 14 March 2022, 16 April 2022, 16 April 2022, and 16 April 2022, respectively. Mutation Q556K in ORF1a was present in 5751 genomes overall, classified in several lineages including the B.1.177 lineage (or Nextclade 20A.EU1, or Marseille-2) and some Delta and BA.1 and BA.2 genomes. Mutation K2557R in ORF1b was present in 140,862 sequenced genomes including the Alpha, Delta, BA.1, BA.2 and Eta variants. Mutation A67V was present in 2,294,944 genomes overall, mostly from the Omicron BA.1/21K variant, and from the Eta variant. Finally, mutation A829T in spike was detected in 1351 genomes overall, being particularly present in some Delta variant lineages, mostly AY.39, AY.9.2, and A.6. Only 13 genomes harbored these four mutations concurrently, which were classified as Omicron BA.2 or BA.5 variants: they originate from Israel (*n* = 8, Germany (*n* = 2), or Austria, France, and South Africa (in one case each). Additionally, we found 1411 SARS-CoV-2 genome sequences originating from Seychelles, including 30 from Omicron variants and from samples collected in April 2022, but none were classified as the Omicron BA.5 variant. No clinical sample from the index case’s spouse was available to sequence the SARS-CoV-2 genome. The SARS-CoV-2 isolate could not be obtained from the index patient’s nasopharyngeal sample as it had been inactivated.

### 3.4. SARS-CoV-2 Spike Structure Analyses

**Figure 3:**
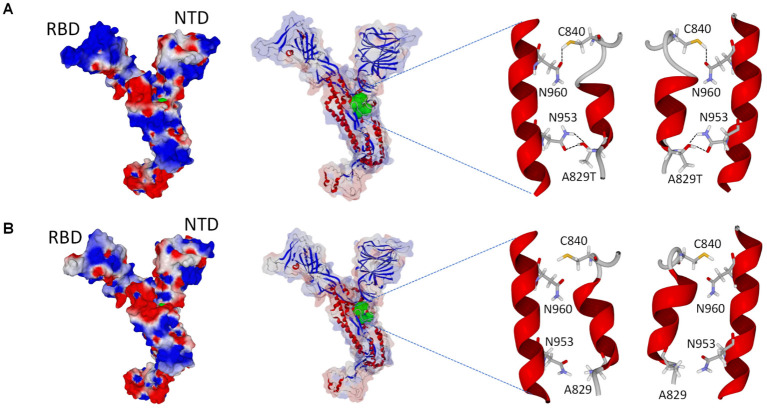
Structural characterization of the BA.5/22B variant. (**A**): BA.5 spike protein; (**B**): original Wuhan spike protein. From left to right: (i) electrostatic surface potential of the whole spike protein; (ii) secondary structure of the spike protein, amino acid residues 829, 840, 953, and 960 are represented in green atomic spheres; (iii) focus on the 829–840 and 953–960 regions of the spike protein, in the case of BA.5/22B, the A829T mutation allows the formation of hydrogen bond network involving C840-N960 and T829-N953 pairs of amino acid residues. This hydrogen bond network is not possible in the original Wuhan spike protein A829. Two opposite faces of the region (front and reverse) are shown for each protein.

The structural analysis of the spike protein of this BA.5/22B variant revealed some molecular characteristics common to most Omicron strains but also some specificities. The common properties included (Figure 3): (i) a flattened N-terminal domain, which has been observed in all Omicron variants, with the noticeable exception of the very first Omicron virus BA.1 whose N-terminal (NTD) domain is more globular [13,28]; (ii) a receptor binding domain (RBD) facing the ACE2 receptor with an increased electrostatic surface potential.

These properties are generally interpreted as a kinetic advantage for both the NTD-ganglioside interaction and the RBD-ACE2 complex formation [32]. The specificity of this particular BA.5/22B variant is the presence of mutation A829T. The side chain of amino acid 829 is located in the central area of the spike protein, at a distance of 33 Å from the residue 614, which plays a key role in the conformational change that demasks the RBD [29]. In the case of A829T, the threonine residue allows the formation of a hydrogen bond network with the side chain of N953. Interestingly, this molecular interaction slightly changes the orientation of the alpha-helices displaying those residues, allowing the formation of a second hydrogen bond between the side chains of C840 and N960. Overall, the result is a compaction of the spike protein in this region, which may affect the conformational plasticity of the spike protein and, thus, the conformational changes controlling the fusion process. To what extent this rearrangement may confer an infectivity advantage by the endocytosis pathway remains to be established, since these hydrogen bonds are not pH-dependent.

## 4. Discussion

The present observation is the first evidence of infection by the Omicron BA.5 variant in the Principality of Monaco. On 20 June 2022, a total of 12,616 SARS-CoV-2 cases and 57 related deaths had been reported in Monaco since the beginning of the pandemic (https://coronavirus.jhu.edu/map.html). The present case is associated with a one-week tourist stay in the Seychelles. Given the incubation period of this infection (mean and median being 6.3 days (range, 1.8–11.9 days) and 5.4 days (range, 2.0–17.9 days), respectively) [33], the occurrence of clinical symptoms the day of the return from the trip suggests that this case is related to the travel abroad. However, it cannot be ruled out that contamination may have occurred during the outward journey or in the hours preceding the outward flight, at the airport, or even in Monaco itself. No other genome of the Omicron BA.5 variant has been deposited in the GISAID database (https://www.gisaid.org/; accessed on 11 July 2022) whose origin is the Seychelles or Monaco; but the number of genomes is only 30 since April 2022 for the Seychelles and zero for Monaco. In the eventuality of contamination during the stay in the Seychelles, this may have occurred though contact with the autochthonous population or with other travelers. This observation is likely an additional example of the difficulty of limiting the circulation of new variants between countries during international transport and of the inadequacy of the measures put in place for this goal. This has been shown very clearly by a recent study which detected the Omicron BA.1 variant in the wastewater of aircraft arriving in Marseille (France) from Addis-Ababa (Ethiopia), while the passengers had been considered uninfected on boarding [15].

The potential of the Omicron BA.5 variant to determine a new epidemic of significant magnitude is currently unknown. Previous variants have had different fates regarding their temporal and geographical spread. A few of them have been labelled as “variants of concern” and have been pandemic (https://www.who.int/activities/tracking-SARS-CoV-2-variants/; accessed on 17 July 2022), whereas others had more limited incidence and restricted spread, such as the Marseille-1, Eta, or Mu variants [34,35,36]. The reasons for these different epidemiological outcomes remain unpredictable. At the stage when variants have been already involved in a substantial number of cases, transmissibility can be estimated, but this might vary according to the age of the epidemic [37,38]. Predictions have been attempted based on nucleotide and amino acid patterns of the spike protein [28] but the level of genetic diversity and its unpredictability makes reliable forecasts tricky. Apart from the spike gene, the Omicron BA.5/22B variant exhibits a D3N substitution in the membrane protein and a D61L reversion in the ORF6 gene. This variant have many spike mutations in common with the Omicron BA.2/21L variant and, to a lower extent, with the Omicron BA.1/21K as well. Binding affinity to ACE2 was lower in the Omicron BA.5/22B variant than in the Omicron BA.1/21K [12]. Substitutions F486V and R493Q (a reversion) in the spike protein have been suspected to be involved in this decrease. In South Africa, The Omicron BA.5/22B and BA.4/22A variants rapidly replaced the Omicron BA.2/21L variant [39]. Estimated growth advantage was 0.12 and 0.08, respectively. Here, the atypical impact of the rarely observed mutation A829T in a BA.5/22B background deserves being followed up.

Finally, these cases show the necessity to adapt constantly the qPCR tests used to screen for variants as their accuracy to predict genome-based classification is hampered by the new variants [19]. As a matter of fact, the variant screening qPCR used, here, did not identify the BA.5/22B variant. Additionally, these cases warrant performing genome-based genotypic surveillance to survey accurately the emergence and circulation of SARS-CoV-2 variants worldwide, whose first occurrence in a country is often with international travel despite some implemented countermeasures. A close monitoring of the emergence and outcome of SARS-CoV-2 variants is valuable because these can have different characteristics regarding transmissibility, pathogenicity, and escape to immune responses elicited by previous infection or by vaccination [37,38], and each determines an independent epidemic [19]. This is particularly of interest to perform, as exhaustively as possible, during periods of low SARS-CoV-2 incidence, to detect new variants as early as possible. During periods of high incidence, to save time and money, it is possible to test only a determined sample of specimens from SARS-CoV-2-diagnosed patients while maintaining reasonable sensitivity for the detection of new variants.

## Data Availability

The genome sequence obtained and analyzed, here, was deposited in the NCBI GenBank nucleotide sequence database (https://www.ncbi.nlm.nih.gov/genbank/; accessed on 11 July 2022) (Sayers et al., 2022) [31] (Accession no. ON989858) and on the IHU Méditerranée Infection website (https://www.mediterranee-infection.com/tout-sur-le-coronavirus/sequencage-genomique-sars-cov-2/; accessed on 11 July 2022; no. IHUCOVID-080068).
